# The impact of estimated glomerular filtration rate equations on chronic kidney disease staging in pediatric renal or heart transplant recipients

**DOI:** 10.1007/s00467-016-3312-x

**Published:** 2016-02-09

**Authors:** Aram Ben Vroling, Eiske Margaretha Dorresteijn, Karlien Cransberg, Yolanda Brigitta de Rijke

**Affiliations:** Department of Clinical Chemistry, Erasmus University Medical Center, Rotterdam, The Netherlands; Department of Pediatric Nephrology, Erasmus University Medical Center–Sophia Children’s Hospital, Wytemaweg 80, 3015 CN Rotterdam, The Netherlands

**Keywords:** Children, Estimated glomerular filtration rate, Chronic kidney disease, Renal transplantation, Heart transplantation, Chemotherapy, Schwartz equation

## Abstract

**Background:**

The aim of this study was to evaluate the performance of selected pediatric estimated glomerular filtration rate (eGFR) equations in relation to the clinical management of children after renal or heart transplantation or post-chemotherapy treatment.

**Methods:**

This study was a retrospective cross-sectional analysis of 61 children whose glomerular function (GFR) had been determined using a single-dose inulin clearance (iGFR) method. Eight equations for estimating the GFR were evaluated for bias, agreement, accuracy, and clinical stratification.

**Results:**

The outcome of all eight eGFR equations differed from the value determined using the iGFR method, with the mean bias ranging from −3.4 to 20.7 ml/min/1.73 m^2^ and 90 % accuracy ranging from 16 to 26 %. All eGFR equations overestimated renal function in patients with decreased kidney function as determined by the iGFR method and underestimated renal function in patients with normal kidney function. Consequently, based on the eGFR values, patients with low GFR values according to the iGFR method were staged in a less severe chronic kidney disease (CKD) category, and patients with normal GFR values according to the iGFR method were staged in a more severe CKD category. The percentage of correctly classified patients ranged from 32.6 to 41.6 %.

**Conclusions:**

In our cohort we found the CKiDIII equation to be the best alternative to calculating the GFR using the inulin clearance method, closely followed by the Hoste and the revised Grubb equations. The performances of all eight eGFR equations assessed were moderate at best and only slightly better than the easy-to-do bedside Schwartz equation.

## Introduction

The ideal reference method for defining glomerular filtration rate (GFR) is measuring the clearance of a marker that is freely filtered in the glomerulus, not metabolized, and not secreted by or re-absorbed in the tubule. The gold standard for calculating the GFR is based on measuring blood levels or urine excretion following a single injection or a steady state infusion of an exogenous marker, such as iohexol, iothalamate, inulin, or ^51^Cr-EDTA, from which the GFR can be calculated [1, 2]. However, these tests are burdensome and expensive, and thus less feasible for routine clinical practice [3]. Consequently, clinicians prefer the alternative, endogenous markers, mostly with the use of serum creatinine-based formulas, validated against reference methods. There are, however, a number of limitations associated with creatinine-based methods, such as the creatinine level being related to muscle mass, creatinine being actively secreted by the proximal tubule in the case of severe renal functional impairment, and creatinine being metabolized extrarenally by intestinal bacteria [4–8].

This has led to growing interest in alternative methods to calculate the GFR, including the use of cystatin C, which was introduced as a marker for glomerular filtration in 1985 [9]. Cystatin C is a 13.3-kDa molecule that is ubiquitously produced by all nucleated cells, freely filtered in the glomerulus, and subsequently completely re-absorbed and degraded in the proximal tubule [10]. The cystatin C production rate per cell is relatively stable throughout life from 2 years of age, and it thereby may be an attractive marker of GFR in both children and adults [10, 11]. It has been described as an almost ideal marker, with only few limitations, such as its extrarenal elimination (though presumed to be negligible), its dose-dependent correlation to the use of glucocorticoids, and the influence of thyroid dysfunction or diabetes mellitus [12].

The considerable body of research which has focused on the development and validation of equations for estimating GFR using creatinine, cystatin C, or a combination of both [13–23] has mainly involved cohorts with specific ranges of age and kidney function. As a consequence, the outcomes of these equations are hard to extrapolate to other populations.

In the study reported here, , we retrospectively compared the performance of eight selected equations used to estimate GFR, all published between 1999 and 2014 and based on serum creatinine and/or cystatin C, with the GFR calculated from a single-injection inulin clearance method, in a cohort of children and adolescents after heart or kidney transplantation, or post nephrotoxic treatment of a malignancy. We also assessed the reliability of the different equations to predict chronic kidney disease (CKD) classification and thereby the clinical management of the individual patient.

## Materials and methods

### Patients and study design

This study is a retrospective, cross-sectional analysis of patients in whom the GFR was assessed by a single-dose inulin clearance method (iGFR). In our tertiary pediatric nephrology center, inulin clearance is a routine test for patients after renal transplant, heart transplant or post nephrotoxic chemotherapy. In renal transplant patients, iGFR is performed yearly in the first 3 years after transplantation and every other year thereafter. In heart transplant patients, it is performed 1 year after heart transplantation and in some cases pre-heart transplantation, while in some oncology patients it is performed at least 1 year after cessation of chemotherapy. Our patient cohort consisted of only patients in whom serum creatinine, urea, and cystatin C were measured concomitantly.

We first performed a PubMed literature search with the MESH terms “infant”, “child”, “adolescent”, “glomerular filtration rate”, “creatinine”, and “cystatin C”, initially selecting cystatin C- and/or creatinine-based GFR equations which had been published during the last 20 years and developed in a pediatric cohort. We then selected the equations in those studies that matched ours in terms of cohort age, range of GFR, and biochemical analysis used. The criteria were: (1) creatinine measurement by enzymatic assay, (2) cystatin C measurement by turbidimetric assay, and (3) the equation had to be developed for use in a pediatric and adolescent cohort with a range of renal function from severely decreased to normal. Studies originally designed for adult cohorts, but whose application was analyzed in pediatric patients, were also included.

### Biochemical analysis

All biochemical analyses were performed on a Hitachi analyzer (Roche Diagnostics GmbH, Penzberg, Germany). Creatinine was measured using the Creatinine Plus version 2 enzymatic assay (Roche Diagnostics), with interassay coefficients of variation (CV) of 1.0 and 0.8 % at 85.7 and 370 μmol/l, respectively. Blood urea was measured using the UREAL assay (Roche Diagnostics), with interassay CV of 2.2 and 1.3 % at 6.88 and 23.7 μmol/l, respectively. Cystatin C was measured using the Tina-quant cystatin C assay (Roche Diagnostics), with interassay CV of 1.9 and 2.5 % at 1.08 and 4.61 mg/l, respectively. The inulin analysis and subsequent GFR calculation were performed as described by van Rossum et al. [24] with blood sampling at 10, 30, 90, and 240 min after injection of 5000 mg/1.73 m^2^ polyfructosan (Inutest 25 %; Fresenius, Linz, Austria). All patients had a single cannula placed for the inulin (polyfructosan) injection, which was thoroughly flushed prior to blood sampling. The inulin clearance was calculated using MW/Pharm 3.5 (Mediware, Groningen, the Netherlands), a pharmacokinetic computer program using a two-compartment model by Bayesian analysis to calculate estimated GFR (eGFR). This Bayesian analysis combines information from the population pharmacokinetic parameters with information derived from the actual individual concentrations of samples to estimate the individual pharmacokinetic parameters. The calculation is based on the following pharmacokinetic parameters: plasma clearance of inulin, volume of distribution of the central compartment, intercompartmental clearance, volume of distribution of the peripheral compartment. Data on these pharmacokinetic parameters are obtained from the continuous infusion of inulin. The interassay CV for the inulin concentration were 6.9 and 1.9 % at 136 and 725 mg/l, respectively. An iGFR of >120 ml/min/1.73 m^2^ was set at 120 ml/min/1.73 m^2^.

### Statistical analysis

Patient data were collected from our hospital data storage unit where patient laboratory results are stored. Statistical analyses were performed with SPSS statistical software (version 22.0; IBM Corp., Armonk, NY) and Analyse-It for Excel ver. 3.91 (Analyse-It Software, Leeds, UK; Microsoft Corp., Redmond, WA). The eGFR calculated with the different equations were compared to the GFR calculated by inulin clearance (iGFR). A repeated-measures analysis of variance (ANOVA) was used to establish whether sequential measurements could serve as individual data points. We used Bland–Altman difference plot analysis to analyze agreement between iGFR and the eGFR determined using the different eGFR equations. This analysis results in a correlation between the two methods and limits of agreement, comprised of systemic (bias) and random (precision) error, where the limits of agreement can be used as a measure of total error [25]. Accuracy was calculated as follows: the difference between the iGFR and each of the eight eGFR outcomes was expressed as a percentage of the iGFR. To compare equations, we calculated the percentage of the samples whose eGFR differed by ≤10 % (±10 %), ≤20 % (±20 %), or ≤30 % (±30 %), resulting in an accuracy of 90, 80, or 70 %, respectively.

We also compared CKD staging based on the different eGFR equations with that based on the iGFR. The National Kidney Foundation Kidney Disease Outcomes Quality Initiative (KDOQI) stages were applied, including the discrimination between stage 3a and 3b, since the difference between these stages can have consequences for treatment and monitoring [26]. These stages are: stage 1, GFR >90 ml/min/1.73 m^2^; stage 2, 60–89 ml/min/1.73 m^2^; stage 3a, 45–59 ml/min/1.73 m^2^ stage 3b,  30–44 ml/min/1.73 m^2^; stage 4, 15–29 ml/min/1.73 m^2^; stage 5, <15 ml/min/1.73 m^2^. The abilities of the different eGFR equations to assign the correct CKD class were compared with a McNemar’s test, which is effectively a chi-square test on a 6×6 contingency table, and the Cohen’s Kappa (κ) was calculated as a measure of agreement [27]. The κ coefficient indicates the proportion of agreement over and above chance agreement, with a κ value of 1 indicating complete agreement and a κ value of 0 indicating no agreement. The* P* value indicates whether the coefficient is statistically significantly different from zero.

## Results

### Study subjects

From February 2009 up to January 2013, 90 inulin clearance tests (iGFR range 13–178 ml/min/1.73 m^2^) had been performed in 61 patients (age range 3.2–19.1 years). Forty-five and eight patients had undergone renal transplantation (70 measurements) and heart transplantation (12 measurements), respectively, two were pre-heart transplantation patients (2 measurements), and six patients (6 measurements) had a history of a malignancy (3 rhabdomyosarcoma, 2 Ewing sarcoma, 1 hepatoblastoma). Since our dataset contained sequential measurements we performed a repeated measures ANOVA analysis to test whether repeated measures would influence the outcome of the analyses. The interactions between the repeated measurements and eGFR outcome were not significant (*P* = 0.281). Descriptive characteristics of the study cohort are given in Table 1.

**Table 1:** Patient characteristics at time of inulin glomerular filtration rate measurement

Descriptive characteristics of patient cohort	Values
Number of samples	90
Number of patients	61
Gender distribution of samples	Male 53 (59 %)
Age (years)	12.5 (7.8–16.4)
Weight (kg)	39.7 (18.5)
Height (cm)	141.5 (23.3)
Post kidney transplant/post heart transplant/other	NTX 70 (78 %)/HTX 12 (13 %)/other 8 (9 %)
Inulin GFR range (ml/min/1.73 m^2^)	13–120
Inulin GFR median (ml/min/1.73 m^2^)	74 (53–97)
CKD stage (number of measurements)	
CKD stage 5 (<15 ml/min/1.73 m^2^)	1 (1 %)
CKD stage 4 (15–29 ml/min/1.73 m^2^)	9 (10 %)
CKD stage 3b (30–44 ml/min/1.73 m^2^)	12 (13 %)
CKD stage 3a (45–59 ml/min/1.73 m^2^)	23 (26 %)
CKD stage 2 (60–89 ml/min/1.73 m^2^)	21 (23 %)
CKD stage 1 (> 0 ml/min/1.73 m^2^)	24 (27 %)

Data are presented as a number (*n*) with/without the percentage in parenthesis, as appropriate, or as the median with the interquartile range (IQR) given in parenthesis

NTX, Post kidney transplant; HTX, post heart transplant; GFR, glomerular filtration rate; CKD, chronic kidney disease

### Selection of eGFR equation

Our literature search identified 20 cystatin C- and/or creatinine-based GFR formulas that had been developed or validated in children (Table 2). Of these 20 studies, seven (with 8 eGFR equations) met our criteria of comparable biochemical analysis, cohort age, and GFR distribution: Schwartz et al. (modified bedside and CKIDIII) [23], Zappitelli et al. [31], Hoste et al. [29], Grubb et al. (original and revised) [21, 22], Filler et al. [33], and Bökenkamp et al. [19]. The Schwartz modified bedside, Hoste, and Zappitelli equations are creatinine based, the Grubb original and revised, Filler, and Bökenkamp equations are cystatin C based, and the Schwartz CKiDIII is both creatinine and cystatin C based.

**Table 2:** Studies in literature in which an estimated glomerular filtration rate equation was developed, validated, and/or analyzed in a pediatric population

Authors/study reference^a^	Year of publication	Biochemical analysis method (manufacturer)	Equation	Cohort age (years)^g^	Cohort GFR (ml/min/1.73 m^2^)
eGFR equation based on serum creatinine only					
** Schwartz et al. (Bedside)** [23]	2009	Enzymatic creatinine assay (Siemens Healthcare)	41.3 × Ht × Scr^−1^	10.8 (7.7–14.3)^g^	41.3 (32.0–51.7)^g^
Pottel et al. [28]	2012	Age <5 years enzymatic creatinine assay (F. Hoffmann-La Roche AG, Basel, Switzerland), Age >5 years Jaffe creatinine assay (F. Hoffmann-La Roche AG)	107.3 × Q^b^ × Scr^−1^	1.6-14.0^f^	11–162.3^f^
**Hoste et al. **[29]	2014	Enzymatic creatinine assay (F. Hoffmann-La Roche AG)	107.3 × Q^c^ × Scr^−1^	0.1–20^f^ (development) 10–25^f^ (validation)	95 (72–112^g^ (validation)
Leger et al. [30]	2002	Jaffe creatinine assay (F. Hoffmann-La Roche AG)	0.641 × weight × Scr^−1^ + 16.063(Ht)^2^ × Scr^−1^	0.8–18^f^	31–200^f^
**Zappitelli et al. **[31]	2010	Enzymatic creatinine assay (Vitros; Ortho Clinical Diagnostics)	inverse ln of: (8.067 + [.034 × ln (0.011/Scr)] + (0.305 × ln age) (+0.064 if male)	12.1 (8.2–15.7)^g^	82 (54–99)^g^
Levey et al. [32]	2009	Enzymatic creatinine assay (F. Hoffmann-La Roche AG)	141 × min(Scr/κ,1)α × max(Scr/κ,1) −1.209 × 0.993Age × 1.018 (if female) × 1.159 (if black)^d^	47 (15)^h^	68 (40)^h^
eGFR equation based on serum cystatin C only					
** Grubb et al. original **[21]	2005	Turbidimetric cystatin C assay (Dako)	84.69 × CysC^-1.68^ (if age <14 years × 1.384)	0.3–17^f^	11–240^f^
** Grubb et al. revised **[22]	2014	7 assays, nephelometric and turbidimetric cystatin C assays (Abbot, Dako, Gentian, F. Hoffmann-La Roche AG, Sentinel and Siemens)	130 × CysC^-1.069^ × age^-0.117^ − 7	12.0 (2.0–17.5)^i^	103 (27–200)^i^
** Filler et al. **[33]	1999	Turbidimetric cystatin C assay (Dako)	91.62 × cysC^-1.123^	1.7–18^f^	12–211^f^
** Bökenkamp et al. **[19]	1999	Turbidimetric cystatin C assay (Dako)	137 × CysC^−1^ − 20.4	0.2–-17.96^f^	7–84^f^
Zappitelli et al. [13]	2006	Nephelometric cystatin C assay (Dade-Behring)	75.94 × CysC^-1.17^, if renal transplant, ×1.2	12.7 (4.7)^h^	74 (36)^h^
Larsson et al. [16]	2004	Nephelometric cystatin C assay (Dade-Behring)	77.24 × CysC^-1.2623^	4–92^f^	8–115^f^
Hoek et al. [14]	2003	Nephelometric cystatin C assay (Dade-Behring)	−4.32 + 80.35 × CysC^−1^	1–-77^f^	12.3–157^f^
Rule et al. [17]	2006	Nephelometric cystatin C assay (Dade-Behring)	76.6 × CysC ^-1.16^	51(15)^h^	57(29)^h^
Le Bricon et al. [15]	2000	Nephelometric cystatin C assay (Dade Behring)	78 × CysC^−1^ + 4	46 (9)^h^	18–76^f^
eGFR equation based on both cystatin C and creatinine					
Bouvet et al. [20]	2006	Jaffe creatinine assay (Olympus), nephelometric cystatin C assay (Dade-Behring)	63.2 × (1.2/CysC)^0.56^ × (1.086/Scr)^0.35^ × (weight/45)^0.30^ × (age/14)^0.40^	1.4–21.4^f^	18–198^f^
Zappitelli et al. [13]	2006	Enzymatic creatinine assay, (Vitros), nephelometric cystatin C assay (Dade-Behring)	43.82 × (CysC)^-0.635^ × (Scr)^-0.547^ × (1.35^Ht^)	12.1 (8.–-15.7)^g^	82 (54–99)^g^
**Schwartz et al. (CKiDIII) **[23]	2009	Enzymatic creatinine assay (Siemens), Turbidimetric cystatin C assay (Dako)	39.1 × (Ht/Scr]^0.516^ × (1.8/ CysC)^0.294^ × (30/BUN)^0.169^ × (1.099)^male^ × (Ht/1.4)^0.188^	10.8 (7.7–14.3)^g^	41.3 (32–0-51.7)^g^
Chehade et al. [34]	2014	Jaffe creatinine assay (Roche), nephelometric cystatin C assay (Siemens)	0.42 × Ht/Scr - 0.0004 × (Ht/Scr)^2^ − 14.5 × CysC + 0.69 × age + K^e^	3.9–18.5^f^	16–142^f^

eGFR, estimated GFR (calculated using one of the listed formulas); Scr, Serum creatinine (mg/dl); CysC, serum cystatin C (mg/dl); BUN, serum urea (mg/dl); Ht, height (m) except in Q for the Hoste equation where it is centimeters; weight, in kilograms; age, in years

^a^Study listed in bold reported the equations which we selected for analysis in the present study. Note: CKiDIII is chronic kidney disease in children formula III, where Scr (mg/dL) × 88.4 = μmol//l and BUN (mg/dl) × 0.3570 = μmol/L

^b^Q factor in Pottel’s equation is based on average of age group

^c^Q = 3.94 − 13.4 × height + 17.6 × height^2^ − 9.84 × height^3^ + 2.04 × height^4^ for boys and girls

^d^Cohen’s Kappa (κ) is 0.7 for females and 0.9 for males; α is −0.329 for females and −0.411 for males; min indicates the minimum of Scr/κ or 1; max indicates the maximum of Scr/κ or 1

^e^K = 18.25 for females and 21.88 for males

^f^Values are given as the range

^g^Values are given as the median with the IQR in parenthesis (interquartile range

^h^Values are given as the mean with the standard deviation (SD) in parenthesis

^I^Values are given as the median with the 2.5th to 97.5th percentile given in parenthesis

### eGFR equation performance

**Fig. 1:**
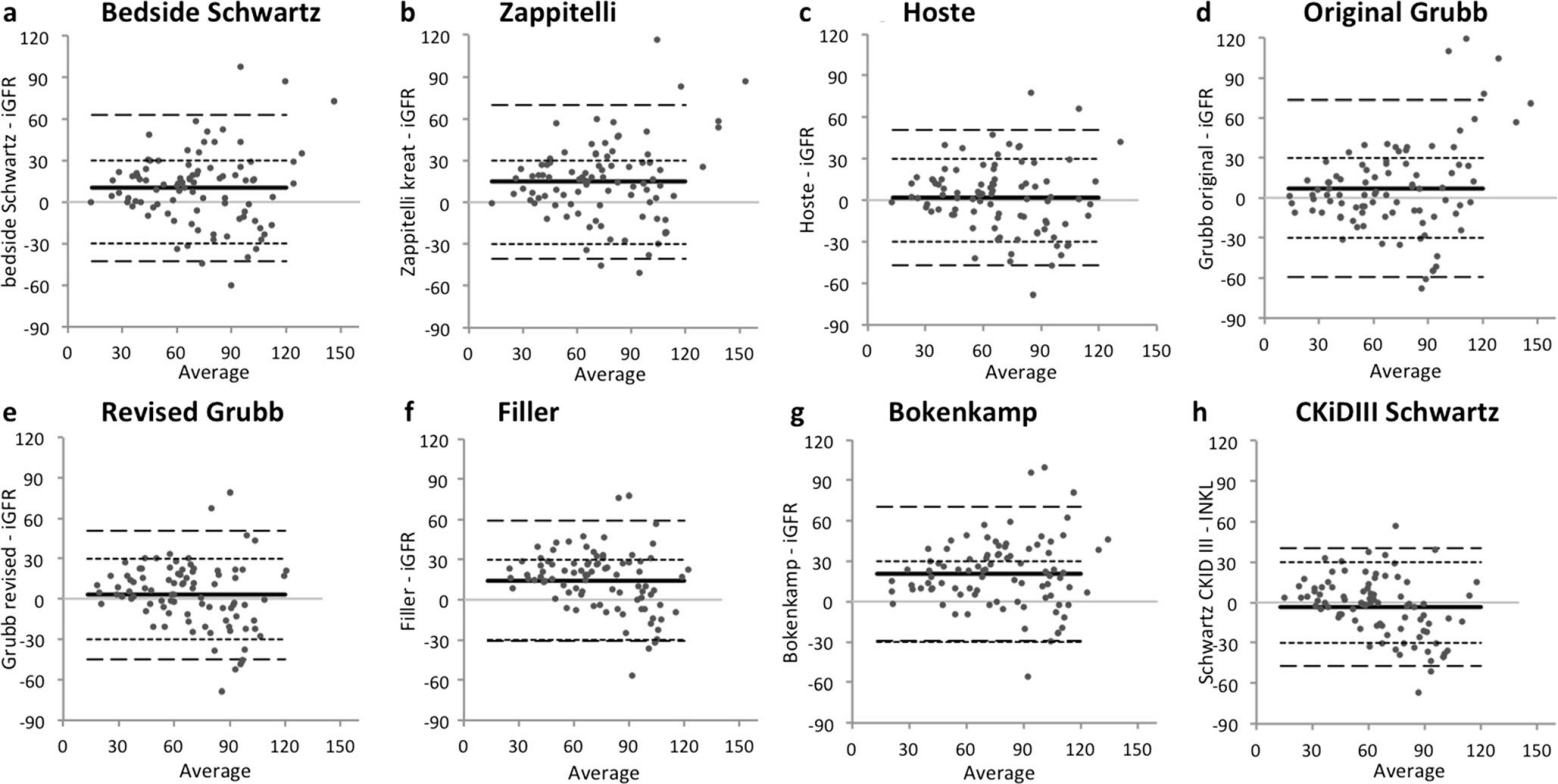
Bland–Altman difference plot analysis between glomerular filtration rate determined by the inulin method (*iGFR*) and estimated GFR (eGFR) as calculated using different equations. GFR is expressed in units of ml/min/1.73 m^2^ *. Horizontal axis* Average iGFR and respective eGFR values,* solid line* mean bias,* broken lines* 95 % limits of agreement,* dotted lines* p30 (line indicating 30 % difference between measurements and average). For chronic kidney disease (CKD) classification, see text (Statistical analysis section) and Table 1

The Bland–Altman difference plot analysis of the iGFR and the eGFRs from the eight equations is shown in Fig. 1, and the bias and limit of agreement values are presented in Table 3. The mean bias of the tested equations ranged from −3.4 to 20.7, with the Hoste [29] equation showing the lowest bias. The original and revised Grubb [21, 22], Hoste [29], and CKiDIII Schwartz [23] equations did not show a significant bias (the 95 % confidence interval of the bias did contain zero) in contrast to the other equations. The smallest interval for limits of agreement, and thereby the lowest total error, was shown by the CKiDIII Schwartz equation [23], followed by the Filler [33] and revised Grubb [22] equations. When we analyzed the performance of the equations using only the data from the renal transplant recipients, we saw a decrease in mean bias, a narrowing of the limits of agreement, and an increase in the coefficient of determination (Table 4).

**Table 3:** Bias and limits of agreement values of Bland–Altman difference plot analysis^a^ between glomerular filtration rate (GFR) determined by the inulin method and the estimated GFR calculated using the different equations

eGFR equations	Bias (95 % CI)	Lower LoA (95 % CI)	Upper LoA (95 % CI)	Coefficient of determination* R* ^2^
Bedside Schwartz [23]	10.0 (4.4–15.7)	−42.8 (−52.5 to −33.2)	62.9 (53.2–72.5)	0.614
Zappitelli [31]	14.6 (8.7–20.6)	−40.6 (−50.8 to −30.5)	69.9 (59.8–80.0)	0.615
Hoste [29]	1.9 (−3.4 to 7.1)	−47.1 (−56.1 to −38.1)	50.9 (41.9–59.9)	0.622
Original Grubb [21]	7.0 (−0.1 to 14.1)	−59.4 (−71.6 to −47.3)	73.4 (61.2–85.6)	0.538
Revised Grubb [22]	3.0 (−2.1 to 8.1)	−44.9 (−53.7 to −36.1)	50.8 (42.1–59.6)	0.619
Filler [33]	14.2 (9.4–19.0)	−30.8 (−39.0 to −22.5)	59.2 (51.0–67.5)	0.655
Bökenkamp [19]	20.7 (15.4–26.0)	−29.2 (−38.3 to −20.0)	70.6 (61.4–79.7)	0.655
CKiDIII Schwartz [23]	−3.4 (−8.1 to 1.3)	−47.2 (−55.3 to −39.1)	40.5 (32.4–48.5)	0.671

LoA, limits of agreement; CI, confidence interval; iGFR, inulin-based GFR

^a^Analysis on whole dataset, including renal, and heart transplant recipients, and oncology patients (61 patients, 90 measurements). Graphical representation is shown in Fig. 1

**Table 4:** Bias and limits of agreement values of Bland–Altman difference plot analysis^a^ between iGFR and eGFR calculated using different equations

eGFR equations	Bias (95 % CI)	Lower LoA (95 % CI)	Upper LoA (95 % CI)	Coefficient of determination* R* ^2^
Bedside Schwartz [23]	6.8 (1.1–12.5)	−39.8 (−49.6 to −30.1)	53.4 (43.7–63.2)	0.678
Zappitelli [31]	11.7 (5.8–17.6)	−36.9 (−47.0 to −26.7)	60.3 ((50.1–70.4)	0.683
Hoste [29]	−0.8 (−6.22 to 4.6)	−45.4 (−54.8 to −36.1)	43.9 (34.5–53.2)	0.675
Original Grubb [21]	2.4 (−3.8 to 8.5)	−48.2 (−58.8 to −37.7)	52.9 (42.4–63.5)	0.668
Revised Grubb [22]	1.0 (−3.9 to 5.9)	−39.5 (−47.9 to −31.0)	41.4 (33.0–49.9)	0.727
Filler [33]	12.3 (7.6–17.0)	−26.4 (−34.5 to −18.3)	51.0 (42.9–59.0)	0.756
Bökenkamp [19]	17.0 (12.1–21.8)	−23.1 (−31.5 to 14.7)	57.0 (48.7–65.4)	0.756
CKiDIII Schwartz [23]	−4.6 (−9.5 to 0.4)	−45.3 (−53.8 to −36.8)	36.2 (27.7–44.7)	0.724

*iGFR* inulin-based glomerular filtration rate, *eGFR* estimated glomerular filtration rate

^a^Analysis on data obtained only from renal transplant recipients (45 patients, 70 measurements)

The difference fit analysis showed that all equations resulted in an overestimation of GFR at low iGFR values and an underestimation of GFR at high iGFR values. Although overestimation is significant in all equations, the effect size varied from 27 (CKiDIII Schwartz [23]) to 47 ml/min/1.73 m^2^ (Filler [33]). All equations showed a negative association between the iGFR and the difference between eGFR and iGFR. The CKiDIII Schwartz [23] equation resulted in the smallest interval for limits of agreement and the highest percentage of samples within 70, 80, and 90 % accuracy (Table 5).

**Table 5:** Accuracy analysis

eGFR equations	70 % accuracy	80 % accuracy	90 % accuracy
Bedside Schwartz [23]	59 %	34 %	19 %
Zappitelli [31]	52 %	33 %	16 %
Hoste [29]	61 %	46 %	21 %
Original Grubb [21]	52 %	38 %	20 %
Revised Grubb [22]	61 %	44 %	21 %
Filler [33]	43 %	34 %	18 %
Bökenkamp [19]	42 %	33 %	19 %
CKIDIII Schwartz [23]	64 %	44 %	26 %

Values in table are the percentages of estimated glomerular filtration rate (eGFR) results per equation that differ by <30, <20, or <10 % from the inulin-based glomerular filtration rate (iGFR), resulting in 70, 80, or 90 % accuracy, respectively

### Clinical stratification by CKD class

**Fig. 2:**
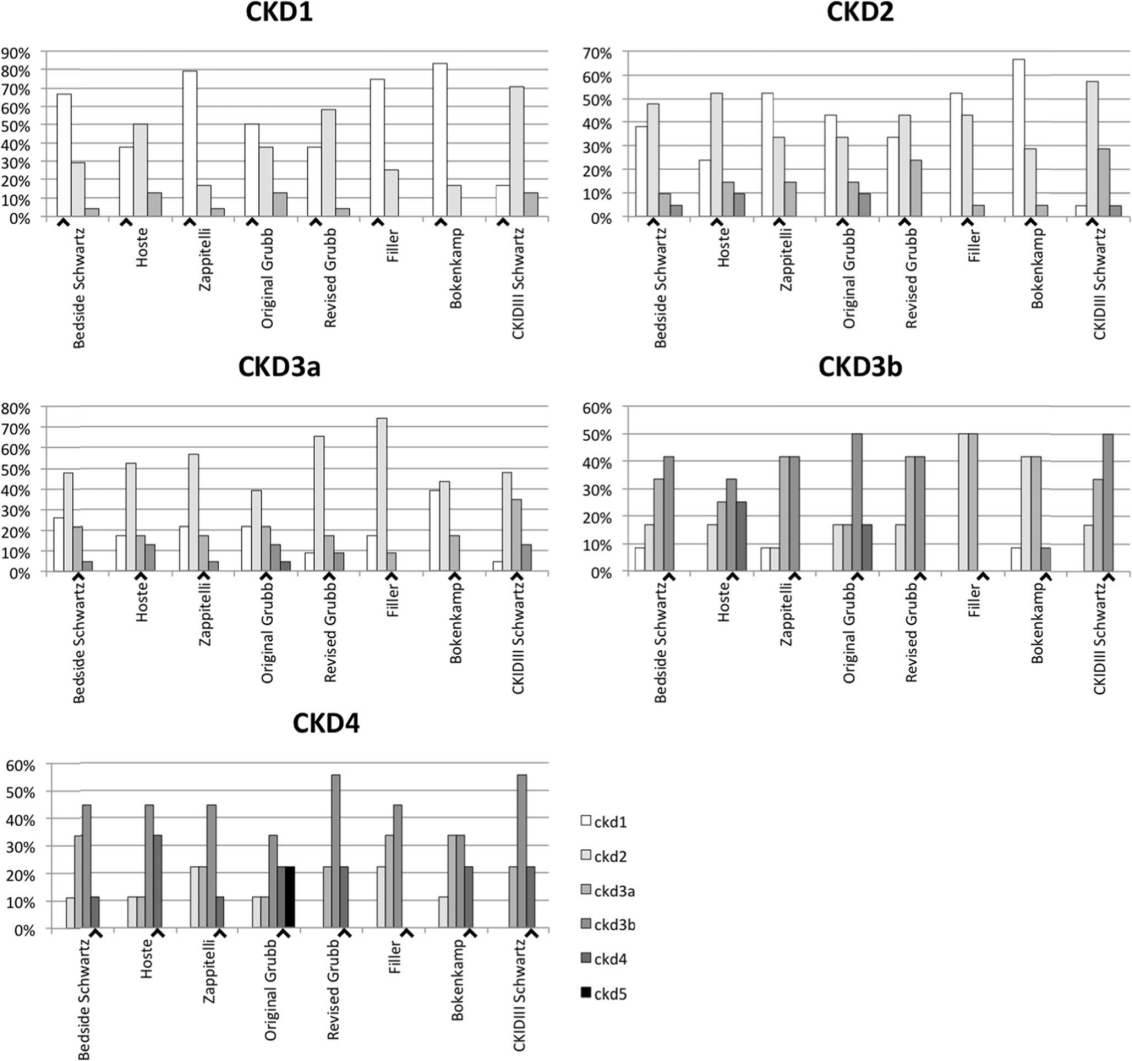
Chronic kidney disease (CKD) classification of patients according to estimated glomerular filtration rate (eGFR) calculated using the different equations, sorted by inulin-based glomerular filtration rate (iGFR)–CKD class. Black arrowheads highlights the correct eGFR class for the iGFR. GFR is expressed in units of ml/min/1.73 m^2^. For CKD classification, see text (Statistical analysis section) and Table 1

Figure 2 shows per iGFR–CKD class the percentage of samples that were assigned to a certain CKD class based on the eGFR. Overall, the eGFR equations resulted in underestimation of CKD stage (overestimation of renal function) compared with the iGFR for patients with decreased iGFR, while the opposite was true for patients with normal GFR. In the group of iGFR–CKD1, the equation of Bökenkamp performed best by correctly classifying 20 of 24 patients, whereas the CKiDIII Schwartz equation correctly classified only four of the 24 patients. The CKiDIII Schwartz equation correctly classified 12 of 20 patients with iGFR–CKD2, but seven patients were staged in a higher (worse) class. In the iGFR–CKD3a and –CKD3b classes, the CKiDIII Schwartz equation resulted in the highest number of correctly classified patients, and in the iGFR–CKD4 group, the Hoste equation resulted in the highest number of correctly classified patients (3 of 9 patients). No results are shown for the iGFR–CKD5 class as only one patient fell into this category.

To test whether classification by the eGFR equation differed from that by iGFR we used a McNemar chi-square test. All* P* values were <0.0001, showing that the eGFR determined by all of the equations assessed were significantly different from the iGFR. To test the agreement between the classifications we performed a Cohen’s κ analysis, the results of which are presented in Table 6. This test showed that agreement overall was poor to moderate. The highest κ value was observed for the bedside Schwartz equation (0.255), suggesting only moderate agreement at best with the CKD classification based on iGFR.

**Table 6:** Summary of chronic kidney disease category analysis

eGFR equations	Percentage correctly classified samples	Cohen’s kappa (κ) (95 % CI)^a^
Bedside Schwartz [23]	41.6	0.255 (0.126–0.384)
Zappitelli [31]	40.4	0.230 (0.103–0.357)
Hoste [29]	34.8	0.176 (0.049–0.303)
Grubb original [21]	36.0	0.177 (0.052–0.302)
Grubb revised [22]	32.6	0.124 (−0.003 to 0.251)
Filler [33]	32.6	0.105 (−0.005 to 0.215)
Bokenkamp [19]	37.1	0.162 (0.046–0.278)
CKIDIII Schwartz [23]	36.0	0.152 (0.036–0.268)

Data are presented as the percentage of correctly classified samples in classes iGFR–CKD1 through to IGFR–CKD4 of all 89 samples in these groups

*CKD* chronic kidney disease, *iGFR* inulin-based glomerular filtration rate, *eGFR* estimated glomerular filtration rate

^a^Cohen’s kappa (κ) analysis of CKD was used to categorize eGFR results (CKD–eGFR) compared to the iGFR (CKD–iGFR)

## Discussion

In this study we addressed the efficacy of eight different equations to estimate GFR based on serum concentrations of creatinine and/or cystatin C compared to the GFR based on inulin clearance in a pediatric cohort of 61 children who were followed after kidney or heart transplantation or after nephrotoxic chemotherapy. Even though the selected GFR equations were matched to our study cohort in terms of age, the GFR range, and the biochemical methods used, the results varied widely, and none of the equations performed particularly well. Even the best coefficient of determination was only* R*^2^ = 0.67, which is very modest given that eGFR and iGFR are supposed to be measuring the same thing and, therefore, are expected to be highly correlated.

Based on the limits of agreement, the CKiDIII Schwartz [23] equation performed best with a LoA interval of 87.8. This equation also showed the highest accuracy with 26, 44, and 64 % of samples differing by <10, <20, or <30 % from the reference method (iGFR), respectively, followed by the Hoste and revised Grubb equations.

With regard to bias the equation by Hoste et al. [29] showed the lowest bias (1.9), followed by the revised Grubb, CKiDIII, and original Grubb equations, with bias of 3.0, −3.4, and 7.0, respectively, all of which were not significant.

Nehus et al. [35] and Bacchetta et al. [36] showed a much better performance of the eGFR equations they investigated in their respective studies, with up to 44 and 48 % of the samples within 90 % accuracy, respectively. This difference from our results may be due to differences in the iGFR range: in the studies of Bacchetta et al. [36] and Nehus et al. [35], the normal range of the iGFR was 100 ± 32 ml/min/1.73 m^2^ [mean ± standard deviation (SD)] and 95 (76–110) ml/min/1.73 m^2^ [median (interquartile range)]. Both values are considerably higher than the iGFR in our cohort [69 (52–94) ml/min/1.73 m^2^] [mean (±SD)]. The inclusion of adolescents in our cohort, in contrast to the CKiDIII cohort, may have decreased the performance of the CKiDIII Schwartz, which in the study of Nehus et al. [35] did perform better than in our study. Therefore, the difference in renal function between the study cohorts in these studies and our cohort likely explains the difference in performance.

An interesting finding of our study is that those equations which performed best analytically (CKiDIII, Hoste, and revised Grubb equations) were not the equations which performed best in terms of CKD classification. This can be explained by the unequal distribution of the number of patients over the different CKD classes. When only patients in CKD classes 3a, 3b, and 4 were analyzed, the CKiDIII, Hoste, and revised Grubb equations successively had the highest number of correctly classified patients.

In our study, we saw that there was a trend for all equations, both cystatin C- and/or creatinine-based, toward overestimating renal function at lower iGFR values and underestimating it at higher iGFR values. This trend was also reflected in CKD staging: when we used the eGFR to assign a CKD stage to every patient we observed an overall tendency to stage patients at a lower (better) class based on their eGFR than on their iGFR. In contrast, in patients with a higher iGFR, renal function will be underestimated, although this will not affect CKD staging, as for most equations this point is ≥90. In general practice, this overestimation of renal function and incorrect CKD classification can cause delay in treatment, over-dosage of medication, and/or inadequate follow up.

The mathematical manipulations in the eGFR equations assessed could potentially explain the difference in performance between them. In the equations based only on cystatin C, the result of the cystatin C assay had a stronger effect on the outcome of the original Grubb equation [21] than on that of the Filler equation [33]. In the revised Grubb equation [22], age has become an exponential factor, whereas in the original Grubb equation it was a dichotomous factor; this change possibly results in a better correlation to the iGFR. The CKiDIII Schwartz equation [23] is very complex because apart from including the height/creatinine ratio, serum cystatin C level, and blood urea level, it also has height as a separate exponential factor and gender as a factor. By including so many parameters that influence the outcome of this equation, each individual parameter has a smaller effect on the eGFR calculated using this formula than in a simpler equation. As a result, such the CKiDIII Swartz equation would be more resistant to aberrations in the parameters on an individual patient level, which can explain why in our study it outperformed the other equations. On the other hand, the simplicity of the bedside Schwartz equation makes it a useful tool in daily practice.

The study by Hoste et al. [29] shows the importance of height correction in eGFR, as was also highlighted by Schwartz [37] and confirmed in studies by Rink et al. [38] and de Souza et al. [39]. All of these studies show that height correction is particularly relevant in adolescent patients. Our study cohort included adolescents, but we did not analyze specific adolescent and pre-adolescent equations. In these mixed populations the use of height-dependent equations can have additional value. Of the equations included in our study, only the Schwartz bedside equation, the CKiDIII equation, and the Hoste equation use height in the calculation of the eGFR.

The equations analyzed in this study were developed in studies with patient cohorts with suspected or established renal pathology. The cohort in our study also included patients before and after heart transplantation or in their follow-up after chemotherapy. The inclusion of a variety of patients may influence the outcome of the performance analysis. Comparing the performance of the equations in these subgroups to that of the whole study cohort would give more insight into the use of these equations in specific patient groups. However, the subgroups of oncology and heart transplantation were very small in our study, making statistical analysis unfeasible. Analysis of only the renal transplant recipient data showed some improvement in performance for all equations. Consequently, using these equations in patients suffering from renal pathology will yield more reliable results than in other patient groups.

Although much work has been done on pediatric equations using creatinine and cystatin C as biomarkers, we found that in our cohort the performance of such equations was modest at best. Better methods, or better equations, are needed to improve and harmonize this field.

Our study has two major strengths. First, we selected eGFR equations from the literature that had been validated in a cohort comparable to our cohort in terms of age, GFR range, and biochemical methods used. This ensured that only eGFR equations were included that should perform best in our cohort. Second, we related the calculated eGFR to CKD staging, which provided good insight in the clinical consequences of implementing these eGFR equations in daily practice. To our knowledge, this latter analysis has not been done previously for pediatric equations.

One limitation needs to be addressed. We did not use the new International Federation of Clinical Chemistry and Laboratory Medicine (IFCC)-calibrated cystatin C assay. The IFCC reference material was established in 2010. Until recently, almost all studies in the literature have used a non-IFCC calibrated cystatin C assay, as a result of which the cystatin C results can be biased, which will affect the eGFR equation that was developed. This bias can lead to inaccuracy in some equations. An exception is a recently published multicenter study by Grubb et al. [22] in which different IFCC standardized assays were used to determine a new (revised) equation to calculate the eGFR from the cystatin C concentration in blood. The use of an IFCC calibrated Roche assay will result in concentrations that are approximately 15 % lower than those used in our earlier assays (internal communication with Roche Diagnostics). As a result, the eGFR from the Grubb 2014 equation will be 23 % higher. When we corrected for the 15 % difference between our assay and the new IFCC-calibrated assay we found a mean bias of 22.6. A recent study by Eckfeldt et al. did not show a 15 % decrease, but rather a 3.3 % increase in cystatin C results when using the Roche IFCC-calibrated assay, which corresponds with our findings using the revised Grubb equation [40].

In conclusion, in our cohort of pediatric and adolescent patients we found the CKiDIII equation to be the best alternative to the inulin clearance test, closely followed by the Hoste and revised Grubb equations, although the performance of all three equations was only moderate at best. All eight equations assessed showed inconsistency throughout the range of iGFR, overestimating renal function at low iGFR values and underestimating renal function at high ones. Since for daily monitoring of renal function and screening of patients most equations may be too elaborate, the modified bedside Schwartz equation remains an acceptable alternative to the iGFR.
